# Joint decision-making, technology adoption and food security: Evidence from rice varieties in eastern India

**DOI:** 10.1016/j.worlddev.2023.106367

**Published:** 2023-11

**Authors:** Maria Luz L. Malabayabas, Ashok K. Mishra, Valerien O. Pede

**Affiliations:** a College of Public Affairs and Development, University of Philippines at Los Baños, Philippines; b Kemper and Ethel Marley Foundation Chair, Morrison School of Agribusiness, W. P. Carey School of Business, Arizona State University, Mesa, AZ 85212, USA; c International Rice Research Institute, Los Baños, Philippines

**Keywords:** Hybrid rice, Decision-making, Treatment effects, Endogenous switching regression

## Abstract

This study investigates the effect of married couples’ joint decision-making on rice variety selection on rice productivity—a measure of food security. The study uses the 2016 Rice Monitoring Survey and the endogenous switching regression (ESR) method. Results show that rice farms with joint decision-makers (husband and wife) would have higher yields, particularly in households that adopted MRVGen1 (before 1986) rice varieties. Thus, families with joint-decision making have higher food security. Intervention programs for food insecurity should acknowledge and incorporate information regarding the persons responsible for making rice varietal decisions and the characteristics of modern rice varieties to ensure food security.

## Highlights

• Spouses are increasingly making farming-related decisions.
• Join-decision making (husband and wife) on farming is increasingly important.
• Assess married couples’ joint decision-making on rice variety selection and rice productivity.
• Farms with joint decision-makers and wife) would have higher yields when selecting older rice varieties.
• Farm families with joint-decision making have higher food security.

## 1 Introduction

Increased crop productivity through the adoption of modern technologies (improved seeds, synthetic fertilizer, irrigation, and mechanization) is considered one of the greatest legacies of the Green Revolution in South and Southeast Asia (Tsusaka and Otsuka, 2013) and a source of food security. The introduction of modern technologies has increased food security and reduced poverty in developing and emerging economies worldwide (de Graft-Johnson et al., 2014). Rice is one of the crops that benefited from genetic improvement and resulted in estimated economic gains between US$ 296 million-US$ 9.9 billion (Raitzer et al., 2015). In India alone, there are more than 900 modern rice varieties (1975–2010) and around 47 hybrid rice varieties^1^ (1994–2010) released by the government of India (DRD, 2020).

Rice plays a vital role in Indian agriculture as a major supplier of calories in the Indian diet, covering approximately 35% of the total area under food grains (GOI, 2023). Despite the rice varietal developments, there has been noticeable slow productivity growth in food grains in recent decades compared to the early decade of the Green Revolution (Khush, 1999). Eastern India^2^ is one region that experienced slow rice productivity and food insecurity. Two possible reasons may contribute to the low growth in rice productivity. These include a lack of desirable traits of high-yielding rice varieties (Hossain et al., 2003) and adverse effects of climatic conditions (Tsusaka and Otsuka, 2013). For example, Pandey et al. (2007) estimated that drought events between 1970 and 2000 resulted in an average loss in rice production of 5.4 million tonnes or $162 million. This low yield growth will substantially impact the region since rice farming is dominated by fragmented and smaller holdings, lack of irrigation facilities, and frequent adverse climatic conditions. Given the conditions in rice farming states in eastern India, this may trigger the continuing vicious circle of low input-low output agriculture and, consequently, food insecurity and loss of livelihood.

To reduce the variability in farm income and uncertainty in livelihood, male heads of households have sought off-farm or dual employment to increase family income. The labor movement from agriculture has increased the daily nominal wage rate for various farm activities, including plowing, sowing, and rice transplanting.^3^ A report by the International Crops Research Institute for the Semi-arid Tropics (ICRISAT) shows that the nominal wage rate increased 3.6 to 4.2 times during the 2004–2014 period (Bhattarai et al., 2014). The movement to the non-rural sector was further enhanced by government programs like the Mahatma Gandhi National Rural Employment Guarantee Act (MGNREGA),^4^ which has led to labor shortages in agricultural production.

In most developing economies, farming decisions (such as selecting crops, technology, and labor) traditionally have been made by the male household heads (Orr et al., 2016). However, in the absence of male decision-makers, spouses are increasingly responsible for making farming-related decisions. Studies by Datta and Mishra (2011) and Maharjan et al. (2012) reveal that the Indian rice farmers’ income sources have become more diversified in recent years, which has led to significant changes in gender roles within the households. Further, existing studies tend to rely on household heads’ information in analyzing the adoption of technologies (Mehar et al., 2017).

Today, it is more likely than in the past that farming-related decisions are made jointly. Joint decision-making is gaining significant traction in literature (Ibrahim et al., 2012, Aregu et al., 2011). To this end, studies investigating the adoption of technologies focusing on male decision-makers may lead to biased estimates (Agarwal et al., 2013). However, there needs to be more studies on the married couple’s participation in decision-making regarding farming-related activities, especially rice seed varieties in India.

Thus, the objective of this study is twofold. First, to investigate the factors affecting married couples’ participation in the joint decision-making of adopting rice varieties. Second, to assess the impact of the joint decision-making strategy on food security—measured by rice productivity. This paper used the 2016 Rice Monitoring Survey, a large nationally representative household-level survey data than was previously reported from India. Our study contributes to the literature in several ways. First, the study focuses on why married couples choose joint decision-making regarding rice varieties. Since this study is based on observational data, the decision-making strategy choices are not distributed randomly, making the two groups systematically different. The study employs endogenous switching regression (ESR) to account for selection bias and endogeneity (Alene and Manyong, 2007, Mishra et al., 2017, Bairagi et al., 2020. Second, the impact of a joint decision-making strategy on rice productivity is generated using counterfactual estimation. Findings from this study will guide policymakers regarding the implementation of agricultural development strategies, technology uptake, and extension programs. In particular, developing outreach materials appropriate for spouses and their involvement in farming to increase food security (or rice productivity) in the eastern region of India.

## 2 Women’s participation in decision-making

Women’s participation in rice farming is often associated with their share in production labor. In eastern India, women provide labor in nurseries, transplanting, weeding, and harvesting, comprising at least 60% of total rice labor requirements (Pandey et al., 2012). However, it is not always guaranteed that women significantly influence decision-making regarding critical issues about farming and household matters. Behura et al. (2012) and Bagchi and Bool-Emerick (2012) found that while women in eastern India contribute substantial labor, the male household head still makes decisions on which technology or technology practices to adopt. Women are responsible for decisions about the selling of rice production. However, in the absence of male decision-makers, spouses are increasingly responsible for making farming-related decisions. Most of the literature tends to rely on household heads’ information to analyze the adoption behavior and exclude women who are not household heads. Due to its simplicity, focusing on the household head is a standard method used in most existing literature, particularly in the South Asian setting. However, with the changes in the sources of income to nonfarm rural sources among rice farming households, there is evidence of the changing household roles, which result in the high participation of women in decision-making regarding household and farming decisions (Paris et al., 2010).

Most of the studies that examine intrahousehold decision-making are from sub-Saharan Africa and Latin America. There is little evidence of the joint decision-making process between married couples in determining rice varietal choice in India. Though the Indian government has implemented several programs to improve women’s status, it is still necessary to know women’s involvement, particularly in farming decisions. Thus, investigating decision-maker constraints is an effective way to understand household technology adoption (Deerie, 2005), particularly in India’s rice farming as a staple food. The characteristics of women who are not household heads but are involved in making decisions about technology adoption, food consumption, and food security^5^ is missing.

## 3 Conceptual and empirical framework

The rice variety that the farmer adopts is an important factor in increasing rice productivity. This study focuses on the decision strategies of married couples of rice farming households in eastern India. The decision strategy will depend on whether the couple has joint participation in selecting rice varieties or the household head^6^ (husband) is the decision-maker. Since the survey queried married couples on seven farm production-related decisions,^7^ we only considered the joint decision-making regarding selecting rice seed varieties. Each of the couples was asked about their involvement in decision-making. Only couples who answered the question were included in the analysis. We categorized decision-making into the following categories: (1) husband only decides in the presence of the wife; (2) wife only decides in the presence of the husband; (3) both husband and wife participated in determining the choice of a rice variety to be used in the coming season. In our data category, 2 is non-existent. Thus, the joint decision-making regarding rice variety takes a value of 1 when both husbands and spouses choose the rice variety and 0 if the husband solely decides on the rice variety.

Several factors are included in estimating the Probit model, in which the data description is presented in Appendix Table 1. The choice of decision-making strategy follows a random utility maximization framework where the latent variable $J_{i}^{*},$ describes the *i*^th^ household decision strategy on whether the couple jointly participates or only the husband solely decides the rice variety. The latent variable, $J_{i}^{*},$ captures the expected benefits that household i receives by jointly participating and can be determined by the observed attributes X, and unobserved characteristics, ε and expressed as:

(1) $$J_{i}^{*}=\alpha X_{i}+\mu_{i}\;\;\;with\;\;J_{i}=\left\{\begin{array}{c} 1\;if\;J_{i}^{*}>0\\ 0\;Otherwise \end{array}\right)$$

The household i will choose a joint participation strategy in deciding on rice variety if $J_{i}^{*}>0$, or 0 otherwise. The error term is $\mu_{i}$ with mean zero and variance $\sigma^{2}$.

The endogenous switching regression (ESR) is used to analyze the impact of the couple’s joint decision-making strategy on rice yield. This method was developed by Lee (1978). Since then, this method has been used in several empirical studies (Alene and Manyong, 2007, Mishra et al., 2017, Bairagi et al., 2020. Separate outcome equations are specified if the couple has joint decision-making and the husband is the sole decision-maker:

(2) $$Y_{1i}=\alpha_{1}Z_{1i}+\varepsilon_{1i}\;\;if\;J_{i}=1$$

(3) $$Y_{2i}=\alpha_{2}Z_{2i}+\varepsilon_{2i}\;\;if\;J_{i}=0$$

where $Y_{i}$ is the outcome variable (yield) of the *i*^th^ household when using couple’s participation strategy (1 = joint decision-making; 0 = husband sole decision-making), Z is a vector of explanatory variables (farmer and plot characteristics), and α are parameters to be estimated. The outcome variable $Y_{1i}$ when the couple jointly decides on the rice variety while $Y_{2i}$ is observed when the husband solely decides on the rice variety. In using OLS, the estimates $\alpha_{1}$ and $\alpha_{2}$ in Equations (1), (2) will suffer selection bias since the choice of strategy is endogenous. This implies that error terms in Equations (2), (3) will have non-zero expected values (Lee, 1978; Madala, 1983). The error terms in the Probit model (first stage), 1, and 2 are assumed to have a tri-variate normal distribution with mean zero and non-singular covariance matrix, which given as

(4) $$Cov\left(\varepsilon_{1i},\varepsilon_{2i},\mu_{i}\right)=\left[\begin{array}{ccc} \sigma_{\varepsilon 2}^{2} & .2 & \sigma_{\varepsilon 2\mu }\\ . & \sigma_{\varepsilon 1}^{2} & \sigma_{\varepsilon 1\mu }\\ . & . & \sigma_{\mu }^{2} \end{array}\right]$$

where $\sigma_{\mu }^{2}$ is the variance of the error term of the selection equation; $\sigma_{\varepsilon 1}^{2}$ and $\sigma_{\varepsilon 2}^{2}$ are variances of the error terms of the outcome functions in 1 and 2; $\sigma_{\varepsilon 1\mu }$ and $\sigma_{\varepsilon 2\mu }$ are the covariance of $\mu_{i}$, $\varepsilon_{1i}$, and $\varepsilon_{2i}$. According to Maddala (1983), since $Y_{1i}$ and $Y_{2i}$ are not simultaneously observed, the covariance between $\mu_{1i}$ and $\mu_{2i}$ are not defined. Based on the given assumptions, the expected values of $\varepsilon_{1i}$ and $\varepsilon_{2i}$ conditional on sample selection are non-zero:

(5) $$E\left[\varepsilon_{1i}|J_{i}=1\right]=\sigma_{\varepsilon 1\mu }\frac{\phi (\alpha X_{i})}{\Phi (\alpha X_{i})}=\sigma_{\varepsilon 1\mu }\lambda_{1i}$$

(6) $$E\left[\varepsilon_{2i}|J_{i}=0\right]=\sigma_{\varepsilon 2\mu }\frac{\phi (\alpha X_{i})}{1-\Phi (\alpha X_{i})}=\sigma_{\varepsilon 2\mu }\lambda_{2i}$$

where ϕ is a standard normal probability density function and Φ standard normal cumulative functions. The ratio between ϕ and Φ evaluated at $\alpha X_{i}$ is the inverse Mills ratio ($\lambda_{1i}$ and $\lambda_{2i}$ in Equations (5), (6). Substituting $\lambda_{1i}=\frac{\phi (\alpha X_{i})}{\Phi (\alpha X_{i})}$ and $\lambda_{2i}=\frac{\phi (\alpha X_{i})}{1-\Phi (\alpha X_{i})}$ in Equations (2), (3), then the outcome equations can be expressed as

(7) $$Y_{1i}=\alpha_{1}Z_{1i}+\sigma_{\varepsilon 1\mu }\lambda_{1i}+\varepsilon_{1i}\;\;if\;J_{i}=1$$

(8) $$Y_{2i}=\alpha_{2}Z_{2i}+\sigma_{\varepsilon 2\mu }\lambda_{2i}+\varepsilon_{2i}\;\;if\;\;J_{i}=0$$

where $\varepsilon_{1i}$ and $\varepsilon_{2i}$ have zero conditional means. If the estimated $\sigma_{\varepsilon 1\mu }$ and $\sigma_{\varepsilon 2\mu }$ are statistically significant we can reject the null hypothesis (absence of sample selectivity bias). The result suggests that there is evidence of endogenous switching. Since the generated regressors arising from two-stage estimation often result in heteroscedastic error terms $\varepsilon_{1i}$ and $\varepsilon_{2i}$, OLS estimates for Equations (7), (8) will be inefficient (Antle, 1983, Khonje et al., 2018). An efficient method in estimating endogenous switching models uses the full information maximum likelihood ([FIML] Loskin and Sajaia, 2004). The FIML simultaneously estimates the selection and outcome equations to have consistent standard errors. The FMIL estimates are obtained using *movestay* command in STATA (Loskin and Sajaia, 2004). On the other hand, for the model to be identified, exclusion restrictions need to be included. Thus, at least one variable in X, which is not included Z.

The choice of instruments is considered valid if they can influence the selection (joint decision-making strategy) equation but not the outcome (yield). The instruments include access to credit and the differences in the couple’s age (husband-wife). Access to credit among women has been proven to benefit women by increasing household assets and savings (Amin and Becker, 1998), resulting in self-confidence and recognition of her role in the household (Sharma and Varma, 2008). Khush (1999) found that access to credit among households in Bangladesh impacted women’s participation in the household decision-making processes. However, not all credit loans were allocated for the improvement in production. Chavas et al. (2005) found that Gambian farmers who availed of loans from *Osusu*^8^ often used the funds for non-farming-related activities, and only a few were used to purchase inputs and equipment. Only a few have existing agricultural loans, most of which were used for medical and school expenses. Thus, this can be used as an instrument since it may influence participation in decision-making but may not directly determine rice productivity.

The difference in the couples’ age is the second instrument that affects how a decision is made—joint or solo (husband). This represents the power relation between the couple. For instance, Kantor (2003) examines Indian female participants in the home-based garment sector and found that a couple with a large age difference would place more power on the husband. In addition, Schneebaum and Mader (2013) found that smaller age differences initiate a joint decision-making process among married couples rather than one person making critical decisions. The age difference may affect women’s participation in deciding on rice variety but not necessarily the outcome variables (yield).

We use Di Falco et al.’s (2011) falsification test for admissibility by to test for the exclusion restrictions, and the result is presented in Appendix Table 2. Results confirm that the instrumental variables jointly affect participation in joint-decision making or the husband solely decides ($\chi^{2}=19.860;p=0.000)$ but do not affect the outcome variable (yield). Additional tests for the inclusion instrument are presented in Appendix Table 3. The Durbin and Wu-Hausmann (DWH) test for exogeneity is found to be insignificant. To test the identification of the model, the Anderson canonical correlation statistic by Baum et al. (2007) was used, which rejects the null hypothesis that the model is underidentified (${\mathrm{LMstatistic}=21.25,\chi }^{2}p=0.000).$ The Anderson-Rubin’s test by Baum et al. (2007) was used to test for weak instrument robust inference and found to be insignificant ($\chi^{2}=0.58;p=0.746)$.

The above results are used to estimate the counterfactuals and average adoption effects using cross-sectional. The conditionally expected outcome (yield) was computed to generate the average treatment effect on treated (ATT) and average treatment effect on untreated (ATU) with joint decision-making (treated group) and husband decision-making (base group) in actual and counterfactual scenarios (Shiferaw et al., 2014). The conditional expectations for each outcome expectations are the following:

Household with joint decision-making (adopters) (actual):

(9) $$E\left(Y_{1i}\right|J=1)={\alpha_{1}Z}_{1i}+\sigma_{\varepsilon 1\mu }\lambda_{1i}$$

Households with husband (or male operator) decision-making (non-adopters) (actual):

(10) $$E\left(Y_{2i}\right|J=0)={\alpha_{2}Z}_{2i}+\sigma_{\varepsilon 2\mu }\lambda_{2i}$$

Households with husband decision-maker that decided to make decisions jointly (counterfactual):

(11) $$E\left(Y_{2i}\right|J=1)={\alpha_{2}Z}_{1i}+\sigma_{\varepsilon 2\mu }\lambda_{1i}$$

A household with joint decision-makers that decided for a husband to make decisions regarding the choice of rice varieties (counterfactual):

(12) $$E\left(Y_{1i}\right|J=0)={\alpha_{1}Z}_{2i}+\sigma_{\varepsilon 1\mu }\lambda_{2i}$$

Recall that the ATT estimates the effect of participation strategy on the actual yield of households that make decisions jointly. Specifically, the difference between Eq. (10), (12):

(13) $${\mathrm{ATT}}_{\mathrm{JD}}=E\left(Y_{1i}\right|J=1)-E\left(Y_{2i}\right|J=1)=Z_{1i}\left(\alpha_{2}-\alpha_{1}\right)+\lambda_{1i}\left(\sigma_{\varepsilon 1\mu }-\sigma_{\varepsilon 2\mu }\right)$$

The impact on yield for husband decision-makers had they jointly made decisions is estimated using the average treatment effect on the untreated (ATU) is the difference between Eq. (12) and Eq. (10) specifically:

(14) $${\mathrm{ATU}}_{\mathrm{MD}}=E\left(Y_{1i}\right|J=0)-E\left(Y_{2i}\right|J=0)=Z_{2i}\left(\alpha_{1}-\alpha_{2}\right)+\lambda_{2i}\left(\sigma_{\varepsilon 1\mu }-\sigma_{\varepsilon 2\mu }\right)$$

The treatment effects can be further identified through heterogeneity effects (Carter and Milon, 2005). A household with joint decision-makers (actual) may have a higher outcome (yield) than those households with husband decision-makers regardless of their strategic decision but due to other unobservable characteristics. This effect is termed “effect base heterogeneity” (BH) and is defined as:

(15) $${\mathrm{BH}}_{\mathrm{JD}}=E\left(Y_{1i}\right|J=1)-E\left(Y_{1i}\right|J=0)$$

(16) $${\mathrm{BH}}_{\mathrm{MD}}=E\left(Y_{2i}\right|J=1)-E\left(Y_{2i}\right|J=0)$$

Therefore, the BH for a household with a joint decision-maker is the difference between Eq. (9) and Eq. (11), while BH for a husband (male) decision-maker is the difference between Eq. (10) and Eq. (12).

**Fig. A1:**
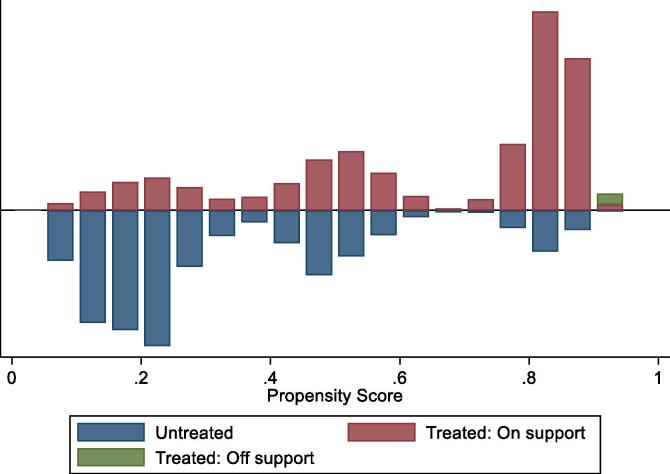
Common support condition.

The robustness of ESR estimates was compared to Propensity Score Matching, commonly used to address selectivity bias due to observable characteristics. This technique matches households with joint and male decision-making based on observable characteristics. Table 2 shows the descriptive statistics of the variables used in the PSM. Appendix Table 5 presents the results of the probit regression, which shows that the spouses’(wife) education and share of assets owned, and the difference in age of the husband and wife has more likely to affect the joint decision-making strategy choice of the household. Appendix Table 6 shows a comparison of two algorithms, shows that the nearest matching (NNM) algorithm with a maximum of three matches with caliper 0.25 $\sigma_{p}$ best suits the analysis. The matching procedure yielded a total of 2,454 matched. A comparison of means was used to assess if there is no significant difference between two groups in the matched sample, thus fulfilling covariates’ balancing condition using pstest command in STATA 17 (Appendix Table 7) (Caliendo and Kopeinig, 2005). A visusal inspection of the overlap in the distribution of the propensity scores shows that the common support is satisfied (Fig. A1). The PSM estimation regarding the effects of yield is presented in Appendix Table 8, which shows that the joint decision-making households have 103.45 kg/ha lower rice yield than male decision-making households with p≥0.548. The results suggest no yield impact regarding who decides the rice variety to plant. In terms of magnitude, the results of PSM estimates are lower compared to ESR estimates. The same results were found by Tesfaye (2016) when comparing the PSM and ESR estimates due to understating the effects leading to a downward bias of results as compared to ESR that accounts for unobserved heterogeneity.

**Table 1:** Sample districts and smallholder households in eastern India, 2016.

State	Number of districts	Number of households
Eastern Uttar Pradesh	37	617
Odisha	30	827
Bihar	16	413
West Bengal	18	614
Total	101	2,471

**Table 2:** Summary statistics of the variables used in the rice variety selection model, Eastern India, 2016.

	Joint decision-maker^1^ households (n = 1,197)	Male decision-maker households (n = 1,274)	All households (n = 2,471)	p-value	
				*t-test*	*Pearson’s ch-sq*
*Dependent variables*					
Yield (kg/ha)	1,545.608	1,679.701	1,614.744	2.242**	
	(10.008)	(1,547.090)	(1,591.461)		
*Explanatory variables*					
Age of the household head^2^ (years)	47.867	48.467	48.176	1.264	
	(11.619)	(11.938)	(11.786)		
Years of education respondent (years)	5.236	6.119	5.691	4.852***	
	(4.228)	(4.779)	(4.541)		
Total number household members	3.515	3.872	3.699	5.446***	
	(1.484)	(1.750)	(1.636)		
Scheduled caste/tribe^3^ (=1 if yes; 0 otherwise)	0.355	0.221	0.286		54.700***
	(0.479)	(0.415)	(0.452)		
Other backward caste^4^ (=1 if yes; 0 otherwise)	0.426	0.396	0.410		2.3666
	(0.495)	(0.489)	(0.492)		
General caste (=1 if yes; 0 otherwise)	0.219	0.384	0.304		79.373***
	(0.414)	(0.487)	(0.460)		
Farm location, Bihar (=1 if yes; 0 otherwise)	0.121	0.370	0.250		204.815***
	(0.326)	(0.483)	(0.433)		
Farm location, Odisha (=1 if yes; 0 otherwise)	0.577	0.107	0.335		613.616***
	(0.494)	(0.309)	(0.472)		
Farm location, West Bengal (=1 if yes; 0 otherwise)	0.257	0.240	0.248		0.969
	(0.437)	(0.427)	(0.432)		
Farm location, Uttar Pradesh (=1 if yes; 0 otherwise)	0.044	0.282	0.167		251.755***
	(0.206)	(0.450)	(0.373)		
Off-farm employment^5^ (=1 if yes; 0 otherwise)	0.766	0.622	0.692		59.740***
	(0.423)	(0.485)	(0.947)		
Share of assets owned by women^6^	22.200	25.755	24.033	3.346***	
	(25.092)	(27.552)	(26.443)		
With migrants^7^ (=1 if yes; 0 otherwise)	0.130	0.149	0.140		1.8135
	(0.336)	(0.)	(0.347)		
Experienced flood/drought 2015 (=1 if yes; 0 otherwise)	0.508	0.632	0.572		38.714***
	(0.500)	(0.482)	(0.495)		
Uses machine (1 = yes; 0 otherwise)	0.795	0.953	0.877		141.643***
	(0.404)	(0.212)	(0.329)		
Uses pesticide (1 = yes; 0 otherwise)	0.444	0.503	0.474		8.772***
	(0.497)	(0.500)	(0.499)		
Transplanted rice (=1 if yes; 0 otherwise)	0.869	0.966	0.919		78.721***
	(0.338)	(0.181)	(0.273)		
Total number of rice plots	1.490	1.301	1.393	−6.703***	
	(0.778)	(0.625)	(0.709)		
Share of irrigated area (%)	40.421	74.520	58.001	19.00***	
	(48.095)	(41.012)	(47.723)		
Proportion of medium land	0.547	0.537	0.550	0.230	
	(0.500)	(0.492)	(0.500)		
Seeds usage (kg/ha)	40.490	36.069	38.211	−2.839**	
	(38.681)	(38.689)	(38.740)		
Total fertilizer (kg/ha)^8^	248.419	294.102	271.972	6.512***	
	(164.251)	(183.184)	(175.725)		
Family labor (person-days/ha)^9^	30.558	30.981	30.776	0.278	
	(33.704)	(41.224)	(37.762)		
Hired labor (person-days/ha)	16.614	15.631	16.107	−1.168	
	(20.517)	(21.252)	(20.901)		
Contract labor (person-days/ha)	10.828	17.981	14.516	6.999***	
	(23.677)	(26.899)	(25.635)		
*Rice varieties*					
Local varieties (TV)	0.129	0.130	0.130		0.015
	(0.335)	(0.337)	(0.336)		
MRVGen1 (before 1986) (=1 if yes; 0 otherwise)	0.077 (0.266)	0.126 (0.332)	0.102 (0.303)		2.887
MRVGen2 (after 1986) (=1 if yes; 0 otherwise)	0.236 (0.425)	0.219 (0.414)	0.227 (0.419)		0.209
				
MRV5 (hybrid rice 1995 and later) (=1 if yes; 0 otherwise)	0.028 (0.164)	0.120* (0.325)	0.075 (0.264)		75.903***
MRV6 (mixed generation) (=1 if yes; 0 otherwise)	0.350 (0.477)	0.231 (0.421)	0.289 (0.453)		42.767***
				
*Instrumental variables*					
With credit^10^	0.316	0.231^***^	0.266		30.108***
	(0.465)	(0.421)	(0.442)		
Difference in age (Husband-wife)	6.045	5.555^***^	5.792	−3.362***	
	(3.420)	(3.802)	(3.630)		
*Logit variables*					
With kids^11^ (=1 if yes; 0 otherwise)	0.479	0.454	0.502		5.670**
	(0.500)	(0.498)	(0.500)		
With extended family^12^ (=1 if yes; 0 otherwise)	0.394 (0.489)	0.358 (0.480)	0.428 (0.495)		12.444***
				
Years of education spouse (wife) (years)	3.296	3.245	3.270	−0.312	
	(3.800)	(4.151)	(3.984)		
Difference years in education (Husband-wife)	1.940	2.872	2.421	6.040***	
	(3.508)	(4.122)	(3.865)		
Total area (ha)	0.455	0.411	0.432	−2.313**	
	(4.341)	(4.060)	(4.200)		

Standard deviations in parentheses: *** p < 0.01, ** p < 0.05, * p < 0.1.

^1^Husband and spouse are making farming-related decisions jointly.

^2^The respondents are the household head who are knowledgeable in rice farming. In this study, all the household heads are husbands.

^3^Includes designated groups of historically disadvantaged indigenous people in India. The terms are recognized in the Constitution of India (GoI), and the various groups are designated in one of the categories. Since independence, the scheduled castes and tribes were given Reservation status, guaranteeing political representation.

^4^Includes castes that are socially and educationally discriminated.

^5^At least one of the household members has off-farm labor like a salaried job (government or service industry) and small business.

^6^Share of productive assets solely owned by women.

^7^At least one member is a migrant.

^8^Total chemical fertilizer used in rice production: NPK- nitrogen, phosphorus and potassium (15–15–15); DAP - diammonium phosphate (18–44-0); and Urea (46–0-0) (http:\https://www.yara.com).

^9^This includes family labor, hired labor, and contract labor. Person-days/ha is the same as person-days/ ha in which 6 h = 1 day.

^10^Credit are for farm and nonfarm purposes.

^11^The children age is 9 years old and below.

^12^Extended family members include parents, siblings, and relatives.

## 4 Survey data

**Fig. 1:**
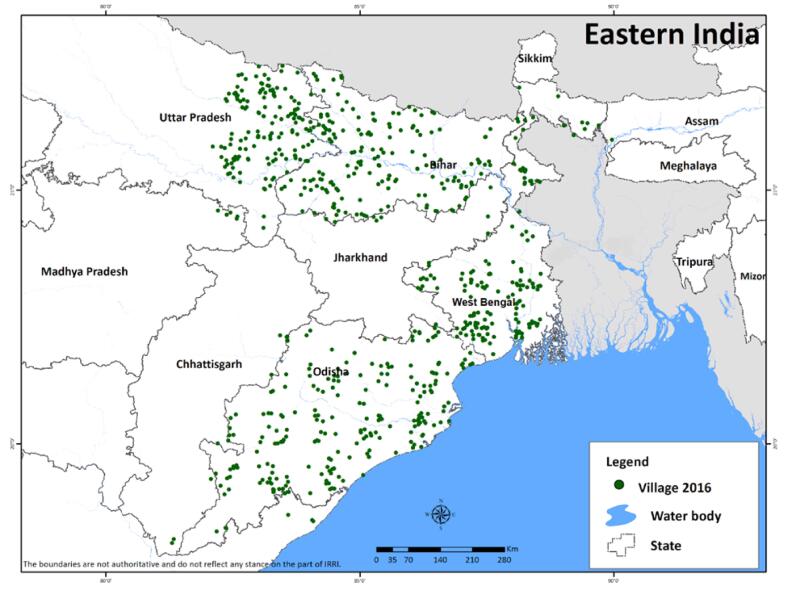
Sample sites in Eastern India.

The study uses the 2016 Rice Monitoring Survey, which focuses on rice farms in eastern India. A rice-producing household is defined as a household that produced rice during the past 12 months. The survey targeted the rural population of eastern India by randomly selecting rural areas based on the 2011 Census of India. Four states in the eastern part of India are considered in the study: eastern Uttar Pradesh, Odisha, Bihar, and West Bengal (Fig. 1). A multi-stage sampling technique was adopted in selecting the respondents. In the first stage, the number of districts was randomly selected in each state using the Census of 2011.^9^ On the other hand, the second stage involves determining the number of villages based on the proportion of each state’s total rice area, keeping the total number of villages at 720. Among the selected villages, household samples are randomly selected using the household census village data. A total of 101 districts and 2,471 rice-producing households are included in the survey (Table 1). Only families that reported with married couple are included in the study. All households in the sample considered the husband as their household male head and wife as their female head. A structured questionnaire was used to interview two household members. Information regarding rice production and farm-related decision-making were collected from husbands, and information regarding livestock, household assets, and farming decisions were also collected from the wife (spouse). The survey employed male and female enumerators in the interview process to elicit unbiased responses. The male enumerator interviewed the household head, while the female enumerator interviewed the spouse. The study focused on information regarding the 2015 wet season, the primary rice-growing season in eastern India.

The summary statistics and definitions of the variables are presented in Table 2 and Appendix Table 1, respectively. The identified household head of all the samples is the husband (male) with an average of 48 years old. Regarding choosing the rice varieties, results show that half of the sample follows a husband decision-making strategy in selecting the varieties. A husband under husband decision-making strategy has higher educational attainment than joint decision-makers. Regardless of decision-makers, it shows that there is a higher number of scheduled tribe/caste (36%) and other backward caste (OBC) (43%) in joint decision-making households than in husband decision-making households. There is a considerably higher number of households under joint decision-making in Odisha and Uttar Pradesh than in male decision-making households. Aside from rice production, respondents have other sources of income or off-work like employment in the private sector and small businesses. Results show that more households with joint decision-making have off-farm work than husband decision-making households. Regarding asset ownership, it shows that spouses (wife) under the husband decision-making households have a higher share of farm asset ownership than in joint decision-making households.

The households in our sample are considered marginal rice farmers, with an average rice area of 0.43 ha. Families under joint decision-making have larger rice areas than households under male decision-makers. Rice farmers allocate at least one plot for rice production during Kharif (wet) season to mostly medium the land part of the landholdings. More than half of the respondents (58%) use supplemental irrigation such as shallow or deep tube wells, implying that some farmers still rely on rainfall as their primary source of irrigation. Since the rice ecosystem in the sample location is considered rainfed, the farmers encounter several water-related problems. In 2015, around 57% of the farmers experienced floods and droughts, particularly in households with husbands’ decision-makers. Recall that Pandey et al. (2007) note that one reason for the low adoption of technology in eastern India is the frequent flooding and droughts, thus hindering productivity.

The rice yield in the sample is lower than the reported national average yield of 3,700 kg/ha (IRRI, 2019). Male decision-making households have significantly higher yield by 134 kg/ha than joint decision-making households. Rice farmers use several significant inputs, including seeds, labor, fertilizer (NPK, DAP, and Urea), machinery, and pesticides. The households under joint decision-making apply significantly larger amounts of seeds than their counterparts. Regarding fertilizer, the households with the husband decision-maker use more fertilizer than joint decision-making households by 46 kg/ha. Regarding machinery and pesticide application, the table shows that a significantly higher number of husband decision-making households use these inputs than their counterparts. Table 2 also presents small-scale rice producers’ labor (family, hired, and contract) requirements. Results show that most households employ the same amount of labor except for contract labor. Families with husband decision-makers use significantly higher contract labor than joint decision-maker households by 7 (persons per day/ha).

Finally, the adoption of rice variety varies depending on the major decision-maker. It shows a significantly higher adopter of MRV5 and MRV6 (mixed) among husband decision-makers and joint decision-making households, respectively. Clear distinctions regarding the two household types are very evident in rice variety adoption. For instance, Gauchan et al. (2012) and Behura et al. (2012) found that farmers use multiple rice varieties depending on the land types that vary according to topographical sequence and moisture level to ensure production that will supply their family consumption needs. Since the sample area is flood/drought-prone, MRV6 (mixed generations) serves as a safety net for household production since most households produce rice for home consumption.

## 5 Results and discussion

The results of the ESR model are presented in Appendix Table 4. Due to brevity and space constraints, we only discuss the effects of the impact model. Table 3 shows the expected quantity of rice produced (kg/ha) under actual and counterfactual conditions. For instance, the cells (A) and (B) represent the expected rice yields (kg/ha) observed in the sample. Cells (C) and (D) represent the expected rice yields (kg/ha) in the counterfactual case. Since there is no selection effect^10^ for the husband as decision-maker, we only focus on the expected rice yield of the joint decision-maker and its counterfactual. In the case of “all rice varieties,” results show that the expected rice yield of households under joint decision-making was about 830 kg/ha and 1,029 kg/ha for husband sole decision-making households. However, this simple comparison could be misleading in attributing the different values of expected yields for both groups to joint decision-making. Columns 5 and 6 of Table 3 report the treatment effects of participation in joint decision-making about variety selection. In the counterfactual case (C), joint decision-making households would have produced less (about 160 kg/ha, or 24%) if they had not adopted a joint decision-making strategy. Similarly, in the counterfactual case (D), rice farmers who did not adopt joint decision-making adopted (or husband decision-maker households) would have produced about 81% less if they had adopted joint decision-making.

**Table 3:** Average treatment effect on treated/untreated and heterogeneity effects for rice yield (kg/ha), by rice variety.

Rice seed variety/generation		Joint decision-making households yield (kg/ ha)	Male decision- making households yield (kg/ ha)	ATE/ATU	
				Change	%
All Varieties	Joint decision	829.69^A^	669.58^C^	160.11***	24
		(21.13)	(14.21)	(25.46)	
	Male decision	192.82^D^	1,029.12^B^	−836.29***	−81
		(4.54)	(20.52)	(21.02)	
	Heterogeneity	668.36***	−384.44***	1,052.80***	
		(48.10)	(44.43)	(47.58)	
Local varieties	Joint decision	739.37	560.24	179.12***	32
		(46.27)	(21.50)	(51.03)	
	Male decision	210.73	838.66	−627.93***	−75
		(13.07)	(35.28)	(37.62)	
	Heterogeneity	528.64***	−278.42***	807.05***	
		(46.58)	(42.08)	(53.60)	
MRVGen1	Joint decision	790.96	567.12	223.85***	39
(before 1986)		(34.23)	(17.02)	(38.23)	
	Male decision	166.43	767.04	−600.61***	−78
		(6.59)	(19.93)	(20.99)	
	Heterogeneity	624.53***	−199.93***	824.46***	
		(32.39)	(26.70)	(37.87)	
MRVGen2	Joint decision	483.71	446.24	37.46	8
(1986 and older)		(26.07)	(20.29)	(33.04)	
	Male decision	120.21	702.46	−582.25***	−83
		(6.09)	(31.82)	(32.40)	
	Heterogeneity	363.49***	−256.22***	619.72***	
		(26.50)	(37.92)	(38.66)	
MRV5	Joint decision	1,243.25	1,125.47	117.79	10
(hybrid rice 1995 and later)		(104.47)	(153.73)	(185.86)	
	Male decision	210.06	1,914.57	−1,704.50***	−89
		(6.67)	(74.08)	(74.38)	
	Heterogeneity	1,033.19***	−789.10***	1,822.29***	
		(50.15)	(174.78)	(176.59)	
MRV6	Joint decision	1,044.06	880.98	163.08***	19
(mixed generation)		(43.34)	(29.42)	(52.38)	
	Male decision	267.97	1,313.84	−1,045.87***	−80
		(12.92)	(47.16)	(48.90)	
	Heterogeneity	776.10***	−432.86***	1,208.96***	
		1,044.06	880.98	163.08***	19

Standard errors in parentheses: *** p < 0.01, ** p < 0.05, * p < 0.1.

Note: ^A^ and ^B^ represents expected yield (kg/ha) observed in the sample.

^C^ and ^D^ represent expected yield (kg/ha) in the counterfactual case.

Conversion: 1 tonne = 1000 kg.

In terms of adopted rice varieties, Table 3 shows that yield advantage differs depending on rice varieties. Most rice smallholder households show a yield advantage when adopting joint decision-making in all rice varieties except for MRVGen2 (1986 and older) and MRV5 (Hybrid). In other words, joint decision-making on rice variety selection positively impacts rice yields for most of the rice variety types particularly MRVGen1 (before 1986). Table 3 (Panel 3) show that the expected rice yield of households producing MRVGen1 under joint decision-making was about 791 kg/ha and 767 kg/ha for husband sole decision-making households. In the counterfactual case, joint decision-making households would have produced less (about 224 kg/ha, or 39%) if they had not adopted a joint decision-making strategy. Similarly, in the counterfactual case (D), rice farmers who did not adopt joint decision-making adopted (or husband decision-maker households) would have produced about 78% more if they had adopted joint decision-making.

Indeed, the study shows exciting findings. A study by Paris et al. (2008) found that male and female farmers in eastern Uttar Pradesh have preferred traits in choosing a particular variety based on varying factors (e.g., environmental, socio-economic, and cultural gender roles). Results showed that farmers prefer rice varieties based on agronomical traits (e.g., yield, tolerance to submergence, pest resistance, and fertilizer responsiveness). In contrast, spouses like the intrinsic qualities of varieties (e.g., taste, cooking qualities, and grain shape). Indeed, results underscore the importance of rice quality when farmers choose rice variety, especially during the joint-decision-making process. Our findings extend the literature findings (Mottaleb, Rahut, and Mishra, 2017) that argue for innovating rice varieties with consumer preferences in mind. However, both men and women farmers prefer high yielding, good taste and aroma, and postharvest quality. One popular mega-variety is Swarna, which covered almost 30% of the total rice area in eastern India in 2015 (Tsusaka et al., 2015) and belongs to the MRVGen1 category. High productivity and consumer preference may be driving factors. MRVGen1 contains attributes that are attractive to both farmers and spouses. For instance, studies (Tsusaka et al., 2015, Mehar et al., 2017) have shown that farmers prefer most mega-varieties due to their higher yield and good eating quality. A sensory evaluation analysis by Champagne et al. (2010) indicates that Swarna has a rough after-cooking surface suited for the thick sauce prominent in Indian cuisine. Thus, it is no surprise that the study found positive effects of joint decision-making on rice yields in MRVGen1—rice varieties bred for grain yields, grain quality, and consumer preference attributes.

Finally, Table 3 reveal transitional heterogeneity effects in adopting a joint decision-making strategy in most of rice variety types. Results indicate that smallholder rice producers with joint decision-makers produced significantly higher rice yields (or more food secure) than households in the counterfactual case (C). Thus, findings show some essential heterogeneity sources that make the rice farming smallholders under joint decision-makers better producers than their counterparts.

The yield advantage also differs depending on the caste where the family belongs (Table 4). For example, households under Other Backward caste (Table 4, panel 2) shows that in counterfactual case (C), joint decision-making households would have produced less (about 113 kg/ha, or 27%) if they had not adopted a joint decision-making strategy. Similarly, in the counterfactual case (D), rice farmers who did not adopt joint decision-making adopted (or male decision-maker households) would have produced about 83% less if they had adopted joint decision-making. The participation of women decision-making among the marginalized are becoming more evident. A study by Paris et al. (2008) found that women from marginalized groups in Eastern Uttar Pradesh are now more empowered, enabling them to choose the rice variety depending on their needs. Results show a transitional heterogeneity effect in adopting a joint decision-making strategy in all caste groups. Results indicate that smallholder rice producers with joint decision-makers produced significantly higher rice yields (or more food secure) than households in the counterfactual case (C).

**Table 4:** Average treatment effect on treated/untreated and heterogeneity effects for rice yield (kg/ha), by caste.

Rice seed variety/generation		Joint decision-making households yield (kg/ ha)	Male decision- making households yield (kg/ ha)	ATE/ATU	
				Change	%
Scheduled tribe/ Scheduled caste	Joint decision	896.083^A^	721.723^C^	174.360***	22
		(38.263)	(25.266)	(45.852)	
	Male decision	227.722^D^	1,106.166^B^	−878.444***	80
		(12.166)	(39.066)	(40.917)	
	Heterogeneity	820.367***	−444.369***	1,264.736***	
		(39.922)	(54.051)	(58.962)	
Other Backward Caste	Joint decision	666.534	548.538	117.997***	27
		(24.783)	(17.429)	(30.298)	
	Male decision	147.758	757.321	−609.562***	−83
		(4.536)	(28.388)	(28.748)	
	Heterogeneity	668.361***	−384.442***	1,052.804***	
		(48.096)	(44.433)	(47.581)	
General	Joint decision	1,039.578	820.602	218.976***	24
		(52.534)	(34.367)	(62.776)	
	Male decision	219.211	1,264.971	−1,045.760***	−79
		(7.908)	(35.013)	(35.895)	
	Heterogeneity	518.776***	−208.783***	727.559***	
		(25.335)	(33.223)	(36.033)	

Standard errors in parentheses: *** p < 0.01, ** p < 0.05, * p < 0.1.

Note: ^A^ and ^B^ represents expected yield (kg/ha) observed in the sample.

^C^ and ^D^ represents expected yield (kg/ha) in the counterfactual case.

Conversion: 1 tonne = 1000 kg.

## 6 Conclusions and implications

This study analyzed the factors affecting joint decision-making in selecting rice varieties and assessed the impact of joint decision-making on food security measures—rice yields. The study used a large and unique 2016 Rice Monitoring Survey and the ESR method. In general, findings showed that farming households with joint decision-making tend to have higher rice yields than their counterparts, but not all the time. Households with joint decision-making strategies positively impacted rice yield, particularly when adopting MRVGen1 (before 1986) varieties. Farmers who adopted joint decision-making tend to have higher rice yields of MRVGen1 (before 1986) than farmers who did not adopt a joint decision-making strategy in the counterfactual. Adoption of MRVGen1 (before 1986) among joint decision-making households increase rice yield due to familiarity and grain quality preference with the varieties. Similarly, a joint decision-making strategy on choosing rice variety has higher yield advantage than male decision-making households in all caste.

Since joint decision-making households perform well when choosing MRVGen1 rice varieties (composed of rice varieties like Swarna,^11^ Lalat, MTU-1001, Moti, and Sambha Mahsori), increasing awareness about the flood-tolerant, better grain quality and improved pest resistance rice varieties should be targeted in the above group. There are ways of expanding a spouse’s participation in rice production activities. First is the involvement in varietal development. One way of verifying newly developed rice variety lines’ acceptability is through Participatory Varietal Selection (PVS). In this method, husband and their spouses can participate in the initial screening before varieties are released (Paris et al., 2008, Paris and Abedin, 2011). Usually, participants are selected based on the proportion of the male-headed and female-headed in the area responsible for making farming decisions in the household. Since our sample shows that eastern India is mainly composed of male-headed families, there is a possibility that women can also participate in choosing rice varieties. For example, Manzanilla et al. (2013) show that female farmers are as knowledgeable as male farmers in evaluating the lines/variety of visible characteristics.

To incorporate women’s participation in PVS strategies for submergence tolerant varieties in Southeast Asia, researchers involved the participating households’ wives by selecting only a sub-sample of the farmer participants (Paris et al., 2011). In India, where most are male-headed households, exploring the individual and the couple’s preferences when selecting new variety lines is warranted. Thus, international research institutes like the International Rice Research Institute (IRRI) should include the preferred grain quality traits in the breeding programs, ultimately benefiting resource-poor smallholders.

Second, there is a high yield impact among joint decision-making strategies when adopting local varieties. Until now, there is a large proportion of farmers that still adopt local varieties despite an increased number of MRV releases in India. This paper defines local varieties as rice varieties that do not have seed certification from the government. It may be the case that the reported local varieties are unrecognizable among farmers due to their continuous use and poor seed systems that affect the rice variety’s authenticity. Tsusaka et al. (2015) found that India’s average varietal replacement age ranges from 18 to 19 years, meaning that farmers will continuously use the same rice variety that usually comes from their previous harvest. To assess the identity of the local varieties, DNA fingerprinting should be explored for more reliable and accurate identification of variety types. A study by Kretzschmar et al. (2018) found that using DNA fingerprinting on the farmer-reported rice varieties in Bangladesh shows that the farmer-reported local rice varieties come from two predominant matches: BR22 (Bangladesh rice variety) and Horidhan (Indian rice variety). Proper identification of these farmer-reported rice varieties will help rice breeders and policymakers accurately assess the impact of rice variety on rice productivity in India.

Third, targeting a woman’s self-help group (WSHG) is one of the most natural pathways to reaching spouses. WSHGs serve as channels for disseminating information, particularly in areas hard for extension workers to enter and marginalized groups are majorly located. Since MRVs are composed of specific agronomical characteristics (e.g., potential yield, grain size, resistance to pests and diseases), information can be disseminated through farmer’s field schools or demonstration plots. The demonstration trials would enable women particularly in the marginalized sector to be exposed to new rice varieties, labor-saving technologies, and proper farm management practices, leading to higher adoption rates.

The current study provided evidence from a large sample of Indian rice farmers. But, the study could be used by policymakers in other developing countries in South and Southeast Asia that are major rice producers. These countries have a similar profile regarding smallholder agriculture, rice production, and livelihood strategies. This study examined the impact of joint decision-making on rice yields, but it has a caveat. The study used cross-sectional data for one rice season, suggesting that the findings only apply on a short-run basis and should be interpreted accordingly. One needs to investigate this issue with panel data to capture long-term adoption impacts. The degree of the spouse’s control within the joint decision-making framework is worth exploring in future studies.

## Data availability statement

Database and fieldwork reports are available by request. Requests to access the datasets should be directed to the corresponding author.

## Ethics statement

Ethical review and approval for the study on human participants was in accordance with the local legislation and institutional requirements.

## Source of funding

We would like to thank all Funders who support this research through their contributions to the CGIAR Trust Fund: https://www.cgiar.org/funders/. In particular, funding from the Stress-tolerant Rice for Africa and South Asia (STRASA) project, the Rice Monitoring Survey (RMS) Project, the Accelerated Genetic Gain in Rice (AGGRi) Alliance project, and from the CGIAR Initiative on Market Intelligence is greatly acknowledged.

## CRediT authorship contribution statement

**Maria L. Malabayabas:** Conceptualization, Methodology, Data curation, Formal analysis, Software, Validation, Writing – original draft. **Ashok K. Mishra:** Writing – review & editing, Visualization, Investigation, Supervision, Validation. **Valerien O. Pede:** Resources, Visualization, Writing – review & editing.

## Declaration of Competing Interest

The authors declare that they have no known competing financial interests or personal relationships that could have appeared to influence the work reported in this paper.

## Figures and Tables

**Table A1:** Variable definition used in the analysis.

Variables	Definition
Male/husband	The terms male and husband are interchangeably used in the study.
Female/spouse/wife	The terms female, wife, and spouse are interchangeably used in the study.
Respondent	Household head and knowledgeable in rice production.
Age (years)	The age of the husband/wife (years)
Education level (years)	The years of education of husband/wife (years)
Difference in years of age of the husband and wife	This is the difference between the husband and wife’s age.
Household size	Number of adults in the house (16 years and above).
Joint decision-making	The participation of men and women: (1) husband and wife jointly participate in deciding the rice variety; (0) men solely decides the rice variety in the presence of the wife.
Share of productive assets solely owned by women (%)	Share of productive assets which are solely owned by women. The productive assets include animals, farm equipment, small and large durables (e.g., TV, refrigerator, and radio).
Caste	These are designated groups of historically marginalized indigenous people in India. The terms are recognized in the Constitution of India (GoI), and the various groups are designated in one of the categories. Since independence, the scheduled castes and scheduled tribes were given reservation status, guaranteeing political representation. There are three caste categories included in this study: Schedule caste and Schedule tribe, Other Backward caste, and General caste.
With off-farm employment	At least one of the household members with off-farm labor like a salaried job, business, and works in the service industry. (=with off-farm employment (1), otherwise 0)
With migrant	A migrant is a person who is away from home for at least one continuous month during the last 12 months to search for a job or to work in another village, district, state, or country. If at least one member is a migrant (1), otherwise (0)
Share of irrigated area	Share of irrigated rice area to the total rice area (%).
Proportion of mediumland	This is the proportion of area that a farmer considers mediumland to the total rice area. Mediumland is a land that is between the lowland and upland.
Experienced flood/drought 2015	This indicates if the farmer experienced flood, drought, or both in cropping the year. (1 = yes, 0 otherwise) 2015
Use machine	Machine used in rice production (e.g., wheel tractor, thresher, seeder, and irrigation pump). (1 = used machine, 0 otherwise)
Seeds use (kg/ha)	Seeds used in kg/ha
Fertilizer use (kg/ha)	Total chemical fertilizer used in rice production: NPK- nitrogen, phosphorus and potassium (15–15–15); DAP - diammonium phosphate (18–44-0); and Urea (46–0-0).
Total plots	Total number of plots the household is cultivated for rice.
Labor	Labor use can be classified as hired labor (person-days/ha); family labor (person-days/ha); and contract labor (person-days/ha). 1 day = 6 h
Rice variety types^1^	
MRVGen1 (before 1986)	This group of rice varieties was released before 1986. Rice varieties that were released before 1976 are characthigher yieldingr-yielding than local varieties and responsive to fertilizer. The varieties released from 1977 to 1985 are resistant to pests and diseases and yields are very stable. The popular varieties in this group are the following: Mahsuri (1972), Annapura (1976), and Swarna (1979).
MRVGen2 (released after 1986 but exclude	This group is considered to have better grain qualities, particularly the one released in 1986–1995. In the later period, the varieties are designed for adverse environments. The popular varieties in this group are the following: MTU-1010 (1995), Moti (1988), Pooja (1999), and Swarna-Sub1 (2009)
MRV5 (Hybrid rice varieties)	These varieties are first-generation offspring of crossbred rice varieties between two genetically diverse parents resulting in higher yield (Barcla 2007). The popular varieties in this group are the following: Arize 6444 (2015), PHB71 (1997), and GK 5000 (2008) Hybrid rice varieties (HRV) refer to all first-generation offspring of crossbred rice varieties between two genetically diverse parents resulting in higher yields (
MRV6 (Mixed)	Mixture of MRVGen1 and MRVGen2 but does not include MRV5 (Hybrid).
Local varieties	Varieties that farmers used during 2015 but do not have a seed certification from the government.
Additional variables used for PSM	
With kids (1 = yes;0 otherwise)	Households with kids 9 years or younger.
With extended family (1 = yes;0 otherwise)	Households living with extended family members like parents, parents-in-law, siblings, and cousins)
Years of education spouse (wife)	The years of education of the wife (years)
Difference in the education (husband-wife)	This is the difference between the husband and wife’s education.
Total rice area (ha)	Total area allocated for rice (ha)

Source: Laborte et al., 2015, Launio et al., 2008 and GOI (2023).

**Table A2:** Parameter estimates-test on the validity of selection estimates.

	Model 1	Model 2
	Joint decision-making (1/0)	Total yield (kg/ha) with Male decision making
Age of the respondent^2^ (years), log	0.031	−0.053
	(0.141)	(0.196)
Years of education respondent^2^ (years), log	−0.002	0.003
	(0.004)	(0.006)
Total household members, log	−0.016	−0.285**
	(0.085)	(0.113)
Scheduled caste/tribe^3^ (=1 if yes; 0 otherwise)	0.172**	0.054
	(0.082)	(0.117)
Other backward caste^4^ (=1 if yes; 0 otherwise)	0.388***	−0.082
	(0.079)	(0.104)
Farm location, Bihar state (=1 if yes; 0 otherwise)	0.631***	0.670***
	(0.117)	(0.132)
Farm location, Odisha state (=1 if yes; 0 otherwise)	2.389***	−0.912***
	(0.145)	(0.208)
Farm location, West Bengal state (=1 if yes; 0 otherwise)	1.545***	0.237
	(0.144)	(0.178)
Off-farm employment^5^ (=1 if yes; 0 otherwise)	0.075	0.117
	(0.075)	(0.094)
Share of women ownership in productive assets^6^	0.005***	−0.007***
	(0.001)	(0.002)
With migrants^7^ (=1 if yes; 0 otherwise)	0.236**	0.190
	(0.091)	(0.094)
Experienced flood/drought2015 (=1 if yes; 0 otherwise)	−0.142**	−0.089
	(0.066)	(0.092)
Uses machine (1 = yes; 0 otherwise)	−0.188	−0.061
	(0.111)	(0.211)
Uses pesticide (1 = yes; 0 otherwise)	−0.169**	−0.086
	(0.067)	(0.092)
Share of irrigated area (%)	0.001	−0.002
	(0.001)	(0.001)
Proportion of medium land	0.171**	−0.349***
	(0.064)	(0.091)
Transplanted rice (=1 if yes; 0 otherwise)	0.157	−0.455*
	(0.122)	(0.251)
Total number of rice plots	−0.008	−0.041
	(0.085)	(0.134)
Seeds usage (kg/ha), log	0.031	−0.174**
	(0.053)	(0.071)
Total family labor (persons day/ha)^8^, log	−0.018	0.083***
	(0.016)	(0.018)
Total hired labor (persons day/ha)^8^, log	0.001	−0.010
	(0.007)	(0.009)
Total contract labor (persons day/ha)^8^, log	−0.021***	0.021**
	(0.007)	(0.010)
Total fertilizer (kg/ha)^9^, log	−0.060	0.104
	(0.064)	(0.082)
MVGen1 (before 1977––1985) (=1 if yes; 0 otherwise)	0.002	−0.040
	(0.093)	(0.130)
MVGen 2 (1985 and later) (=1 if yes; 0 otherwise)	−0.062	0.132
	(0.112)	(0.151)
MRV5 (hybrid rice 1995 and later) (=1 if yes; 0 otherwise)	−0.113	0.512***
	(0.148)	(0.169)
MRV6 (mixed generation) (=1 if yes; 0 otherwise)	−0.082	0.819***
	(0.150)	(0.223)
Constant	−1.556**	7.657***
	(0.621)	(0.868)
Instrumental variables		
With credit^10^ (=1 if yes; 0 otherwise)	0.253***	−0.025
	(0.066)	(0.100)
Difference in age^11^ (Husband-wife)	−0.021**	0.009
	(0.009)	(0.012)
Validity test		
Wald test $\chi^{2}$/ or F-stat (Joint test of the instrumental variables	19.860***	0.310
Pseudo-R^2^ / R-squared	0.300	0.210
Number of observations	2,471	1,274

Standard errors in parentheses: *** p < 0.01, ** p < 0.05, * p < 0.1.

^1^Husband and spouse are making farming-related decisions jointly.

2 The respondents are the household head who are knowledgeable in rice farming. In this study, all the household heads are husbands.

3 Includes designated groups of historically disadvantaged indigenous people in India. The terms are recognized in the Constitution of India (GoI), and the various groups are designated in one of the categories. Since independence, the scheduled castes and tribes were given Reservation status, guaranteeing political representation.

4 Includes castes that are socially and educationally discriminated.

5 At least one of the household members has off-farm labor like a salaried job (government or service industry) and small business.

6 Share of productive assets solely owned by women.

7 At least one member is a migrant.

8 Total chemical fertilizer used in rice production: NPK- nitrogen, phosphorus and potassium (15–15–15); DAP - diammonium phosphate (18-44-0); and Urea (46-0-0) (http:\https://www.yara.com).

9 This includes family labor, hired labor, and contract labor. Person-days/ha is the same as person-days/ ha in which 6 h = 1 day.

10 Credits are for farm and nonfarm purposes.

11 Children age 9 and under. ^12^ Extended family members include parents, siblings, and relatives.

**Table A3:** Additional test for exclusion restrictions.

Test	Null hypothesis/Test type	Test results
Durbin test	Exclusion instrument is exogenous	$\chi^{2}=0.57;p=0.450$
Wu-Hausman test	Exclusion instrument is exogenous	F = 0.562; p = 0.453
Anderson canonical correlation statistic	Underidentification	LM Statistic = 21.25 $\chi^{2}p=000$
Anderson-Rubin’s	Weak instrument robust test	$\chi^{2}=0.58;p=0.746$

**Table A4:** Parameter estimates of couples’ decision-making strategies and yield (kg/ha) log, endogenous switching regression (ESR) approach.

Variables	(1)	(2)	(3)	
	OLS	ESR		
			Regime 1 (Joint decision-making = 1)	Regime 2 (Joint decision-making = 0)
	Total yield (kg/ha), log	Joint decision-making (1/0)	Total yield (kg/ha), log among joint decision- making couple	Total yield (kg/ha), log among male decision- making couple
Joint decision-making (=1 if yes; 0 otherwise)^1^	−0.012			
	(0.075)			
Age of the respondent^2^ (years), log	0.095	0.040	0.150	−0.012
	(0.142)	(0.140)	(0.213)	(0.185)
Years of education respondent^2^ (years), log	0.002	0.002	0.001	0.003
	(0.005)	(0.004)	(0.007)	(0.006)
Total number household members, log	−0.246**	−0.017	−0.087	−0.296
	(0.089)	(0.085)	(0.138)	(0.111)
Scheduled caste/tribe^3^ (=1 if yes; 0 otherwise)	0.005	0.153*	0.084	0.055
	(0.089)	(0.082)	(0.139)	(0.116)
Other backward caste^4^ (=1 if yes; 0 otherwise)	0.010	0.392***	0.329**	−0.077
	(0.083)	(0.079)	(0.138)	(0.106)
Farm location, Bihar state (=1 if yes; 0 otherwise)	0.546***	0.638***	−0.107	0.678***
	(0.116)	(0.117)	(0.279)	(0.136)
Farm location, Odisha state (=1 if yes; 0 otherwise)	−0.483***	2.446***	0.900**	−0.892***
	(0.153)	(0.146)	(0.373)	(0.302)
Farm location, West Bengal state (=1 if yes; 0 otherwise)	0.672***	1.558***	1.723***	0.264
	(0.145)	(0.143)	(0.334)	(0.204)
Off-farm employment^5^ (=1 if yes; 0 otherwise)	0.194**	0.043	0.269**	0.116
	(0.076)	(0.074)	(0.127)	(0.093)
Share of women ownership in productive assets^6^	0.001***	0.005***	0.004*	−0.007***
	0.004	(0.001)	(0.269)	(0.116)
With migrant^7^ (=1 if yes; 0 otherwise)	0.248***	0.221**	0.356**	0.191
	(0.094)	(0.091)	(0.147)	(0.119)
Experienced flood/drought 2015 (=1 if yes; 0 otherwise)	0.035	−0.152**	0.085	−0.087
	(0.070)	(0.066)	(0.107)	(0.092)
Uses machine (1 = yes; 0 otherwise)	−0.151	−0.187	−0.307**	−0.052
	(0.113)	(0.111)	(0.145)	(0.209)
Uses pesticide (1 = yes; 0 otherwise)	0.169**	−0.161**	0.375*	−0.087
	(0.070)	(0.067)	(0.110)	(0.092)
Share of irrigated area (%)	−0.0002	0.001	0.002	−0.002
	(0.001)	(0.001)	(0.001)	(0.001)
Proportion of medium land	−0.185***	0.185*	0.074	−0.351***
	(0.067)	(0.064)	(0.101)	(0.091)
Transplanted rice (=1 if yes; 0 otherwise)	−0.482***	0.195	−0.356**	−0.457*
	(0.125)	(0.122)	(0.154)	(0.249)
Total number of rice plots	−0.064	−0.017	−0.033	−0.040
	(0.089)	(0.084)	(0.121)	(0.133)
Seeds usage (kg/ha), log	−0.176***	0.020	−0.082	−0.171**
	(0.055)	(0.053)	(0.087)	(0.070)
Total fertilizer (kg/ha)^8^, log	0.158**	−0.052	0.155	0.102
	(0.066)	(0.064)	(0.108)	(0.081)
Total family labor (persons day/ha)^9^, log	0.082***	−0.019	0.014	0.083***
	(0.016)	(0.016)	(0.031)	(0.018)
Total hired labor (persons day/ha)^9^, log	−0.022***	0.001	−0.032**	−0.009
	(0.007)	(0.007)	(0.012)	(0.009)
Total contract labor (persons day/ha)^9^, log	0.008	−0.023***	−0.022*	0.020**
	(0.007)	(0.007)	(0.012)	(0.010)
MVGen1 (before 1986) (=1 if yes; 0 otherwise)	0.003	−0.006	0.007	−0.037
	(0.101)	(0.093)	(0.155)	(0.128)
MVGen2 (1986 and later) (=1 if yes; 0 otherwise)	0.107	−0.065	−0.099	0.137
	(0.116)	(0.111)	(0.175)	(0.149)
MRV5 (hybrid rice 1995 and later) (=1 if yes; 0 otherwise)	−0.176***	0.020	0.804**	0.516***
	(0.055)	(0.053)	(0.316)	(0.167)
MRV6 (mixed generation) (=1 if yes; 0 otherwise)	−0.176***	0.020	0.471**	0.821***
	(0.055)	(0.053)	(0.226)	(0.220)
Constant	−0.176***	0.020***	3.409***	7.539***
	(0.055)	(0.053)	(1.026)	(0.837)
*Instruments*				
With credit^10^ (=1 if yes; 0 otherwise)		0.228***		
		(0.064)		
Difference in age (Husband-wife)		−0.019**		
		(0.009)		
$\sigma_{i}$			1.654***	1.384***
			(0.061)	(0.027)
$\rho_{i}$			0.536***	0.003
			(0.094)	(0.113)

Standard deviations in parentheses: *** p < 0.01, ** p < 0.05, * p < 0.1.

1 Husband and spouse are making farming-related decisions jointly.

2 The respondents are the household head who are knowledgeable in rice farming. In this study, all the household heads are husbands.

3 Includes designated groups of historically disadvantaged indigenous people in India. The terms are recognized in the Constitution of India (GoI), and the various groups are designated in one of the categories. Since independence, the scheduled castes and tribes were given Reservation status, guaranteeing political representation.

4 Includes castes that are socially and educationally discriminated.

5 At least one of the household members has off-farm labor like a salaried job (government or service industry) and small business.

6 Share of productive assets solely owned by women.

7 At least one member is a migrant.

8 Total chemical fertilizer used in rice production: NPK- nitrogen, phosphorus and potassium (15–15–15); DAP - diammonium phosphate (18–44-0); and Urea (46–0-0) (http:\https://www.yara.com).

9 This includes family labor, hired labor, and contract labor. Person-days/ha is the same as person-days/ ha in which 6 h = 1 day.

10 Credit are for farm and nonfarm purposes.

**Table A5:** Results of Probit model.

Variable	Coef	dy/dx	z	$p>\left|z\right|$
With kids (=1 if yes; 0 otherwise)	0.012	0.005	0.200	0.839
With extended family (=1 if yes; 0 otherwise)	−0.034	−0.013	−0.560	0.578
Education wife	−0.025	−0.010	−3.220	0.001***
Share of women ownership in productive assets^6^	0.005	0.002	4.250	0.000***
Difference in age (Husband-wife)	−0.019	−0.007	−2.140	0.032**
Difference in education (Husband-wife)	−0.012	−0.005	−1.440	0.149
Farm location, Bihar state (=1 if yes; 0 otherwise)	0.515	0.203	5.230	0.000***
Farm location, Odisha state (=1 if yes; 0 otherwise)	2.278	0.719	35.440	0.000***
Farm location, West Bengal state (=1 if yes; 0 otherwise)	1.328	0.476	15.370	0.000***
Total rice area (ha)	0.071	0.028	1.100	0.270
Constant	−1.186			

*** p < 0.01, ** p < 0.05, * p < 0.1.

**Table A6:** Standardized Percentage Balance, Pseudo R2, and Final Sample Size Using Different Matching.

Type of Matching	Standardized percentage bias^a^		Pseudo R^2b^		LR-test		Final sample	
	Before Matching	Before Matching	Before Matching	Before Matching	Before Matching	Before Matching	Before Matching	Before Matching
NN (3)	26.4	2.5	0.256	0.002	876.26***	6.95	1,274(0)	1,180(17)
Kernel (0.04)	26.4	2.9	0.256	0.002	876.26***	8.24	1,274(0)	1,179(18)

*** p < 0.01, ** p < 0.05, *0.10.

^a^Standardized percentage balance – mean of the treated (joint decision-making) minus the mean of the control (male decision-making) divided by the average of the square root of the variance of the treated and control samples. Matching is sufficient if standardized bias is below 3% or 5%.

^b^Lower Pseudo R^2^ after the matching, and there is no systematic difference.

**Table A7:** Means before and after matching.

	Before Matching			After Matching			% R bias
	Joint Decision	Male Decision	t-stat	Joint Decision	Male Decision	t-stat	
With kids^1^ (=1 if yes; 0 otherwise)	0.45	0.50	−2.38**	0.46	0.49	−1.57	32.70
With extended family^1^	0.36	0.43	−3.54***	0.36	0.37	−0.19	94.70
Education wife (years)	3.30	3.25	0.31	3.34	3.43	−0.55	−78.30
Share of women ownership in productive assets^2^	22.20	25.76	−3.35***	21.39	22.14	−0.76	78.80
Difference in age (Husband-wife)	6.05	5.55	3.36***	6.07	6.26	−1.40	60.50
Difference in education (Husband-wife)	1.94	2.87	−6.04***	1.93	1.85	0.58	91.30
Farm location, Bihar state (=1 if yes; 0 otherwise)	0.12	0.37	−14.94***	0.12	0.12	0.02	99.90
Farm location, Odisha state (=1 if yes; 0 otherwise)	0.58	0.11	28.56***	0.57	0.57	−0.06	99.80
Farm location, West Bengal state (=1 if yes; 0 otherwise)	0.26	0.24	0.98	0.26	0.26	0.13	86.80
Total_area (ha)	0.46	0.41	2.31**	0.45	0.47	−1.11	57.20

*** p < 0.01, ** p < 0.05, *0.10.

^1^Family with kids 9 years old and below.

^2^Living with parents, siblings, and relatives.

^3^Share productive assets solely owned by women.

**Table A8:** Average rice yield (t/ha) using Propensity Score Matching (PSM).

Matching Algorithm	ATT	Std. err	t-stat	p-value
Nearest neighbor (3) caliper ${{0.25\sigma }_{p}}^{1})$	−103.42	172.023	−0.60	0.548
Kernel (bw = 0.04)	33.42	85.270	0.39	0.695

^1^$\sigma_{p}=\sqrt{\sigma_{0}^{2}+\sigma_{1}^{2}/2}$, where 0 and 1 are standard deviations of estimated propensity scores of the control and the treatment groups, respectively (see Cochran and Rubin, 1973).

## Data Availability

Data will be made available on request.

## Footnotes

1 Hybrid rice varieties (HRV) refer to all first-generation offspring of crossbred rice varieties between two genetically diverse parents resulting in higher yields (Barclay, 2007).

2 Eastern India is composed of the following states: Assam, Bihar, Chhattisgarh, Jharkhand, Madhya Pradesh, Odisha, West Bengal, and Uttar Pradesh.

3 During the 2004–2014 period, the cultivation cost of major crops (paddy and wheat) increased at an annual rate of 10% due to the rise in labor costs. Labor costs account for up to 50% of the total production cost of paddy cultivation.

4 Enacted as the National Rural Employment Guarantee Act of India (2005) is a public policy in India that pays people to seek employment. The wage rate is higher than the daily wages of agricultural workers (Sharma, 2011).

5 Food security is a broad concept. It is a multidimensional function of food availability, food access, food utilization and food stability. Food availability comprises of food production (total output or yield) and crop diversity. See Barrett (2010), Gregory et al. (2005), and Jenkins and Scanlan (2001) for different measures and dimensions of food security.

6 Household head is household member who is responsible in rice farming. In the married couple sample, all the respondents identified household head as the husband.

7 We use the operator interchangeably with husband and spouse as a wife.

8 *Osusu* is local rotating saving and credit association in Gambia (Chavas et al., 2005).

9 This data set contains information about all the districts, villages, towns, and cities in urban and rural India.

10 The estimated correlation coefficient terms ${(\rho }_{i})$ are presented in Appendix Table 4. Results show that there is positive and significant self-selection in both decision-making strategies, which suggests that observed and unobserved factors affect the joint decision-making strategy choice and rice yield. On the other hand, the correlation coefficient for husband decision-maker is not statistically significant, which means that households under joint decision-makers and husband decision-makers will have the same rice yield using the old decision-making strategy.

11 For the past decades, scientists at IRRI have developed Swarna-Sub1, a flood-tolerant rice, by introgression SUB1 QTL to mega-varieties (Swarna) through marker-assisted crossing (Neeraja et al., 2007; Septiningsih et al., 2009).
